# *β*-Sitosterol Protects against Myocardial Ischemia/Reperfusion Injury via Targeting PPAR*γ*/NF-*κ*B Signalling

**DOI:** 10.1155/2020/2679409

**Published:** 2020-03-28

**Authors:** Fengxia Lin, Luhua Xu, Meizhu Huang, Bin Deng, Weiwei Zhang, Zhicong Zeng, Song Yinzhi

**Affiliations:** Department of Cardiology, Shenzhen Bao'an Traditional Chinese Medicine Hospital Group, The Affiliated Hospital of Guangzhou University of Chinese Medicine, Shenzhen 518133, China

## Abstract

Myocardial ischemia/reperfusion (I/R) injury is a clinically severe complication, which can cause high rates of disability and mortality particularly in patients with myocardial infarction, yet the molecular mechanisms underlying this process remain unclear. This study aimed to explore the protective effects of *β*-sitosterol against myocardial I/R injury and to elucidate the underlying molecular mechanisms. Our results showed that hypoxia/reoxygenation (H/R) treatment suppressed cell viability, induced cell apoptosis and reactive oxygen species production, increased caspase-3 and -9 activities, upregulated caspase-3 and -9 protein expressions, downregulated the Bcl-2 protein expression, and reduced the mitochondrial membrane potential. *β*-Sitosterol treatment attenuated H/R-induced cardiomyocyte injury. Moreover, *β*-sitosterol treatment counteracted the inhibitory effects of H/R treatment on the peroxisome proliferator-activated receptor gamma (PPARγ) expression and enhanced effects of H/R treatment on the NF-κB expression in cardiomyocytes. Furthermore, inhibition of PPARγ impaired the protective actions of *β*-sitosterol against H/R-induced cardiomyocyte injury. In the I/R rats, *β*-sitosterol treatment reduced the myocardial infarcted size and apoptosis, which was attenuated by the inhibition of PPARγ. In conclusion, our results demonstrate that *β*-sitosterol protected against *in vitro* H/R-induced cardiomyocyte injury and *in vivo* myocardial I/R injury. The *β*-sitosterol-mediated cardioprotective effects may involve the modulation of PPAR*γ*/NF-*κ*B signalling during myocardial I/R injury. Further studies are required to further explore the clinical application of *β*-sitosterol in the myocardial I/R injury.

## 1. Introduction

Myocardial ischemia/reperfusion (I/R) injury represents a pathologic process characterized by myocardial tissue damage when reperfusion after ischemia fails to restore the functionality of the organs and also induces further myocardial dysfunction and injury [1]. Myocardial I/R injury is a clinically severe complication, which can cause high rates of disability and mortality particularly in patients with myocardial infarction [2, 3]. As far as we know, various factors such as cardiomyocyte apoptosis, activation of autophagy, and inflammatory response are contributed to the development of myocardial I/R injury [4]. Unfortunately, the effective therapy for myocardial I/R injury is still limited. Thus, more effects are required to develop more effective therapies for a better management of I/R injury.

*β*-Sitosterol is a type of phytosterol, which is found in many plants. *β*-Sitosterol exhibits similar structure and biological functions as cholesterol and functions as important molecules in stabilizing the phospholipid bilayers of cell membranes [5, 6]. There is growing evidence implicating the beneficial role of *β*-sitosterol on the cardiovascular diseases. A recent mechanistic study showed that *β*-sitosterol was effective to enhance cellular glutathione redox cycling, which led to reduced oxidant injury in rat cardiomyocytes [7]. The *N. deflersiana* ethanolic extract that contains *β*-sitosterol also exhibited protective actions against isoproterenol-induced myocardial injuries in the rats [8]. In addition, *β*-sitosterol showed protective effects against carbon tetrachloride-induced hepatotoxicity in rats via the enhancing mitochondrial glutathione redox cycling [9]. *β*-Sitosterol could decrease thymocyte damage induced by irradiation via maintaining the stability of the mitochondrial membrane and modulating intracellular redox balance [10]. As far as we know, the role of *β*-sitosterol in modulating myocardial I/R injury remains largely unknown.

In this study, we first determined the protective actions of *β*-sitosterol in the hypoxia/reoxygenation- (H/R-) stimulated H9c2 cell injury and further explored the underlying molecular mechanisms. In addition, the protective effects of *β*-sitosterol on myocardial I/R injury were further confirmed in the *in vivo* animal model.

## 2. Materials and Methods

### 2.1. H9c2 Cell Culture

The rat cardiomyocyte cell line (H9c2) was purchased from Sigma-Aldrich (St. Louis, USA). H9c2 cells were cultured with DMEM (Sigma-Aldrich) supplied with 10% fetal bovine serum (FBS; Sigma-Aldrich). H9c2 cells were kept in a humidified incubator with 5% CO_2_ at 37°C.

### 2.2. Chemicals and H/R Treatment

β-Sitosterol (Sigma-Aldrich) and proliferator-activated receptor gamma (PPAR*γ*) inhibitor GW9962 (Sigma-Aldrich) were dissolved in dimethyl sulfoxide (DMSO) to 500 *μ*M and stored for subsequent use (final DMSO concentration < 0.1%). For the H/R treatment, the H9c2 cells were cultured in the glucose-free DMEM in a hypoxic incubator supplied with 1% O_2_, 94% N_2_ and 5% CO_2_ at 37 °C for 24 h. After 24 h of exposure to the hypoxia condition, cells were subjected to reoxygenation in a water-saturated atmosphere of 5% CO_2_-95% air for 4 h. For the *β*-sitosterol (10, 20, and 50 *µ*M) and GW9962 (30 *μ*M) treatment, H9c2 cells were treated with *β*-sitosterol and/or GW9962 at 1 h before H/R treatment.

### 2.3. Cell Viability Assay

H9c2 cell viability was determined by the Cell Counting Kit-8 (CCK-8) assay kit (Beyotime, Beijing, China). In brief, the H9c2 cells subjected to different treatments were incubated with 10 *μ*l CCK-8 solution for 4 h in the incubator as suggested by the product manual. The cell viability was evaluated by measuring the absorbance at 450 nm using a microplate reader.

### 2.4. Flow Cytometry for Cell Apoptosis Determination

Cell apoptosis of H9c2 cells subjected to different treatments was evaluated using the Cell Apoptosis Detection kit (Sigma-Aldrich) by following the product manual. In brief, the treated H9c2 cells were harvested with trypsinization. After being washed with PBS for 3 × 10 mins, H9c2 cells were incubated with 5 *μl* fluorescein isothiocyanate-annexin V and 5 µl propidium iodide for 15 min in the dark at 37°C. The H9c2 cell apoptosis was analyzed on a flow cytometer (BD Biosciences).

### 2.5. Caspase-3 and -9 Activity Assay

The activities for caspase-3 and -9 in treated H9c2 cells were, respectively, determined by the caspase-3 and -9 activity assay kits (Abcam, Cambridge, USA) according to the product manual.

### 2.6. Western Blot Analysis

Protein samples from H9c2 cells or heart tissues were extracted using RIPA buffer (Sigma-Aldrich). The concentrations of proteins were measured using the BCA method (Sigma-Aldrich). Equal amounts of protein samples were resolved on a 10% SDS-PAGE gel. The resolved protein samples were then transferred to the PVDF membranes. After being washed with TBST for 3 × 10 mins, the membranes were subjected to incubation with 2% bovine serum albumin at room temperature for 1 h. After that, the membranes were incubated with different primary antibodies against caspase-3, caspase-9, Bcl-2, PPAR*γ*, NF-*κ*B, and *β*-actin (Abcam, Cambridge, USA) overnight at 4°C. The membranes were then washed with TBST for 3 × 10 mins before incubating with the HPR-conjugated secondary antibodies for 2 h at room temperature. The western blot bands were visualized using the ECL kit (BioVision, Milpitas, USA) by following the product manual. *β*-Actin was used as the reference control for normalizing the respective protein levels.

### 2.7. Reactive Oxygen Species (ROS) Production Determination

ROS production was determined using the mitochondrial superoxide indicator (MitoSOX Red; Sigma-Aldrich). In brief, H9c2 cells subjected to different treatments were incubated with 5 *µ*M MitoSOX Red for 10 min. After being washed with PBS for 3 × 10 mins, the ROS production level was assessed by measuring the accumulated red fluorescence with the excitation and emission wavelengths at 510 nm and 580 nm, respectively.

### 2.8. Mitochondrial Membrane Potential (MMP) Assay

The H9c2 MMP was determined by the JC-1 MMP assay kit (Cayman Chemical, Ann Arbor, USA) by following the product manual. In brief, the H9c2 cells subjected to different treatments were incubated with 2 *µ*M of JC-1 probe dye for 30 min at 37°C. The fluorescence signal of the monomeric form (green color) and the JC-1-aggregate form (red) was determined by confocal microscopy at the single-cell level. The MMP was determined as the ratio of the green fluorescent intensity to the red fluorescent intensity.

### 2.9. I/R Model and Drug Treatments

The adult SD rats (200–250 g) were obtained from the Animal Laboratory Center of Guangdong Medical University, and the experiments were under the approval of the Animal Ethics Committee of the Affiliated Hospital of Guangzhou University of Chinese Medicine. All the rats were anaesthetized with intraperitoneal administration of 40 mg/kg pentobarbital. After general anaesthesia, an incision was made in the chest, and left coronary arteries were exposed by blunt forceps dissection. For the I/R model group, the left coronary arteries were subjected to ligation for 30 min and reperfusion for 120 min. For the sham group, rats underwent the same procedure except ligation and reperfusion of the left coronary arteries. For the I/*R* + *β*-sitosterol group, I/R rats received intraperitoneal injection of *β*-sitosterol (100 mg/kg) at 1 h before I/R. For the I/*R* + *β*-sitosterol + GW9962 group, I/R rats received intraperitoneal injection of *β*-sitosterol (100 mg/kg) and GW9962 (10 mg/kg) at 1 h before I/R. After I/R procedures, the rats were sacrificed by intraperitoneal administration of 80 mg/kg pentobarbital. The heart tissues were collected for further analysis.

### 2.10. Myocardial Infarction Size (IS) Determination

The collected heart tissues were frozen at −20°C and sliced into 2 mm thick slices. The slices were subjected to stain with 1% triphenyltetrazolium chloride for 20 min at 37°C. The images of the stained heart slices were captured by a digital camera, and the area at risk (AAR) and IS were analyzed using Image Pro software. Myocardial IS was shown as the ratio of IS to AAR.

### 2.11. Terminal Deoxynucleotidyl Transferase dUTP Nick End Labelling (TUNEL) Assay

The analysis of the cell apoptosis in the heart tissues was evaluated by the TUNEL assay kit (Abcam). The paraffin-embedded heart tissues were sectioned into 5 *µ*m thick slices. The slices were deparaffinized and rehydrated followed by incubating with 10 mM protease K for 15 min. After that, the slices were incubated with the TUNEL reaction mixture for 60 min at 37°C in the dark. The TUNEL POD was then used to treat the slices for 30 min. The stained slices were analyzed under a light microscope. Apoptosis index (%) was expressed as the ratio of the number of TUNEL-positive cells to the total number of cells.

### 2.12. Statistical Analysis

The data analysis was performed using the GraphPad Prism (Version 6.0, GraphPad Software, La Jolla, USA). The data generated from the experiments were expressed as mean ± standard deviation. Significant differences between/among respective treatment groups were assessed using unpaired *t*-test or one-way ANOVA followed by Tukey's post hoc test. ^*∗*^*P* < 0.05 was considered to be statistically significant.

## 3. Results

### 3.1. H/R Treatment Suppressed Cell Viability, Increased Cell Apoptosis and ROS Production, and Suppressed MMP in H9c2 Cells

**Figure 1:**
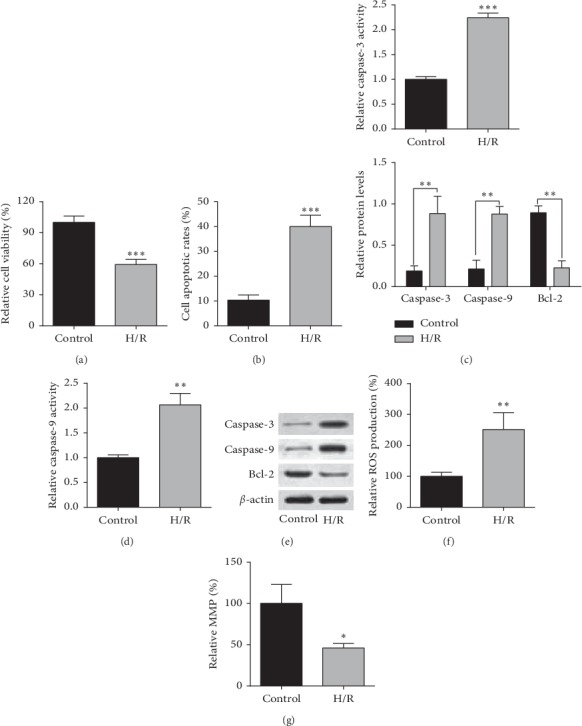
H/R treatment suppressed cell viability, increased cell apoptosis and ROS production, and suppressed MMP in H9c2 cells. (a) Cell viability, (b) cell apoptotic rates, (c) caspase-3 activity, and (d) caspase-9 activity in control H9c2 cells or H9c2 cells subjected to H/R treatment were determined, respectively, by CCK-8, flow cytometry, and caspase-3 and -9 assays. (e) Protein levels of caspase-3, -9, and Bcl-2 in control H9c2 cells or H9c2 cells subjected to H/R treatment were measured by the western blot assay. (f) ROS production and (g) MMP of control H9c2 cells or H9c2 subjected to H/R treatment were determined by ROS production assay and MMP assay, respectively. *N* = 3. ^*∗*^*P* < 0.05, ^*∗∗*^*P* < 0.01, and ^*∗∗∗*^*P* < 0.001.

In the H/R-treated H9c2 cells, the H9c2 cell viability was significantly suppressed as measured by the CCK-8 assay (Figure 1(a)); the H9c2 cell apoptotic rates were elevated as determined by flow cytometry (Figure 1(b)). Moreover, H/R treatment significantly increased the caspase-3 and -9 activities in H9c2 cells when compared to control ones (Figures 1(c) and 1(d)). Western blot analysis showed that H/R treatment increased caspase-3 and -9 protein levels but reduced Bcl-2 protein level in H9c2 cells (Figure 1(e)). Consistently, H/R treatment also enhanced ROS production as well as reduced the MMP in H9c2 cells when compared to control ones (Figures 1(f) and 1(g)).

### 3.2. *β*-Sitosterol Treatment Counteracted H/R-Induced H9c2 Cell Injury

**Figure 2:**
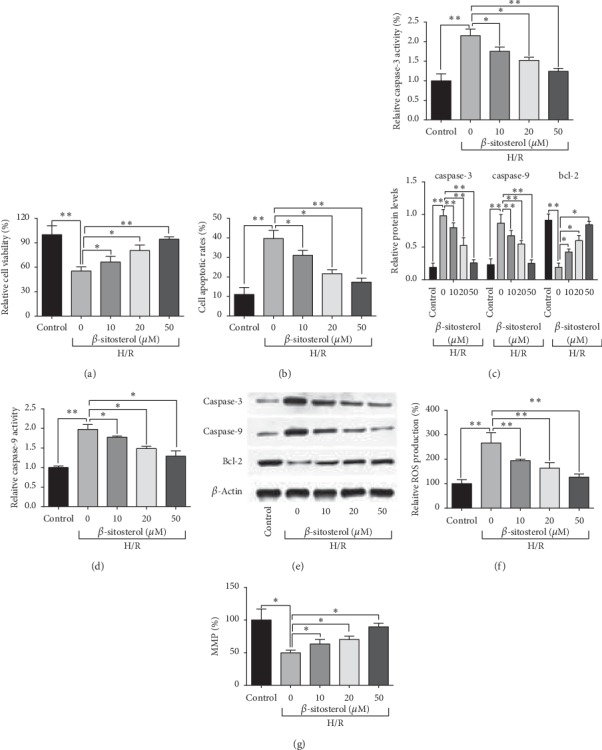
*β*-Sitosterol treatment counteracted H/R treatment-induced H9c2 cell injury. (a) Cell viability, (b) cell apoptotic rates, (c) caspase-3 activity, and (d) caspase-9 activity in H/R-treated H9c2 cells subjected to *β*-sitosterol treatment were determined, respectively, by CCK-8, flow cytometry, and caspase-3 and -9 assays. (e) Protein levels of caspase-3, -9, and Bcl-2 in H/R-treated H9c2 cells subjected to *β*-sitosterol treatment were measured by the western blot assay. (f) ROS production and (g) MMP of H/R-treated H9c2 cells subjected to *β*-sitosterol treatment were determined by the ROS production assay and MMP assay, respectively. *N* = 3. ^*∗*^*P* < 0.05 and ^*∗∗*^*P* < 0.01.

The H/R-treated H9c2 cells were subjected to treatments with elevated concentrations of *β*-sitosterol. As shown in Figures 2(a) and 2(b), *β*-sitosterol treatment concentration dependently increased the cell viability and reduced cell apoptotic rates of H/R-stimulated H9c2 cells (Figures 2(a) and 2(b)). The increase in caspase-3 and -9 activities of H/R-stimulated H9c2 cells was concentration dependently attenuated by *β*-sitosterol (Figures 2(c) and 2(d)); consistently, *β*-sitosterol suppressed caspase-3 and -9 protein levels but increased Bcl-2 protein level in the H/R-stimulated H9c2 cells (Figure 2(e)). In the view of ROS production and MMP, the H/R-induced increase in ROS production and decrease in MMP of H9c2 cells were significantly attenuated by *β*-sitosterol treatment in a concentration-dependent manner (Figures 2(f) and 2(g)).

### 3.3. *β*-Sitosterol Treatment Attenuated the Inhibitory Effects of H/R Treatment on PPARγ/NF-κB Signalling in H9c2 Cells

**Figure 3:**
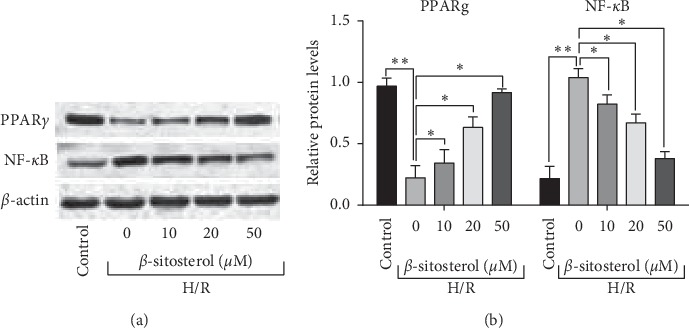
*β*-Sitosterol treatment attenuated the inhibitory effects of H/R treatment on PPAR*γ*/NF-*κ*B signalling in H9c2 cells. Protein levels of PPARγ and NF-*κ*B in H/R-treated H9c2 cells subjected to *β*-sitosterol treatment were measured by the western blot assay. *N* = 3. ^*∗*^*P* < 0.05 and ^*∗∗*^*P* < 0.01.

Furthermore, the western blot analysis showed that H/R treatment significantly suppressed the protein level of PPAR*γ* and increased the protein level of NF-*κ*B in H9c2 cells. *β*-Sitosterol treatment concentration dependently increased PPARγ protein levels but decreased NF-κB protein level in H9c2 cells (Figure 3).

### 3.4. PPARγ Inhibitor Counteracted the Protective Effects of *β*-Sitosterol Treatment against H/R-Stimulated H9c2 Cells

**Figure 4:**
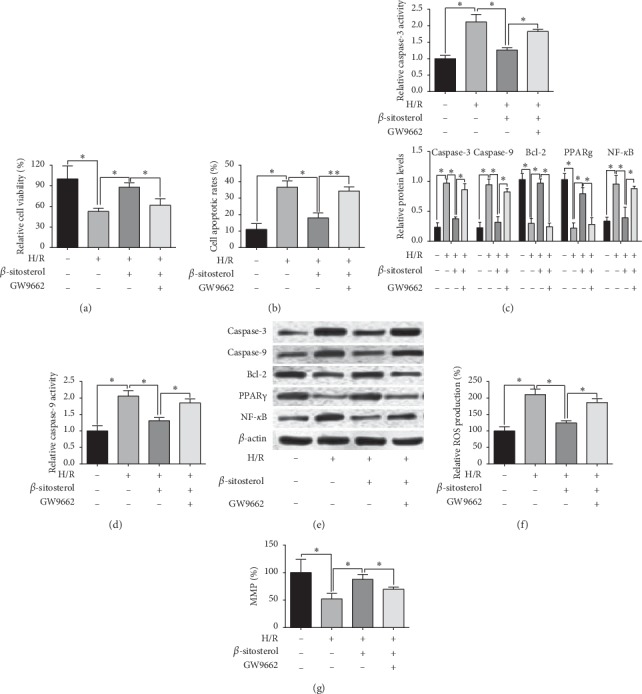
PPARγ inhibitor counteracted the protective effects of *β*-sitosterol treatment against H/R-stimulated H9c2 cells. (a) Cell viability, (b) cell apoptotic rates, (c) caspase-3 activity, and (d) caspase-9 activity in H/R-treated H9c2 cells subjected to *β*-sitosterol and/or GW9962 treatment were determined, respectively, by CCK-8, flow cytometry, and caspase-3 and -9 assays. (e) Protein levels of caspase-3, -9, Bcl-2, PPAR*γ*, and NF-*κ*B in H/R-treated H9c2 cells subjected to *β*-sitosterol and/or GW9662 treatment were measured by the western blot assay. (f) ROS production and (g) MMP of H/R-treated H9c2 cells subjected to *β*-sitosterol and/or GW9962 treatment were determined by the ROS production assay and MMP assay, respectively. *N* = 3. ^*∗*^*P* < 0.05 and ^*∗∗*^*P* < 0.01.

As *β*-sitosterol treatment affected the PPARγ/NF-*κ*B signalling in H/R-treated H9c2 cells, we examined if inhibition of PPARγ could impair the protective effects of *β*-sitosterol on H/R-induced H9c2 cell injury. As shown in Figures 4(a) and 4(b), GW9962 (30 *µ*M) treatment significantly attenuated the *β*-sitosterol-mediated (50 *µ*M) enhanced effects on cell viability and inhibitory effects on cell apoptotic rates of H/R-treated H9c2 cells. In addition, caspase-3 and -9 activities of H/R-treated H9c2 cells were expectedly decreased by *β*-sitosterol treatment, which was attenuated by GW9962 (Figures 4(c) and 4(d)). Consistently, *β*-sitosterol-induced decrease in caspase-3 and -9, and NF-*κ*B protein levels, and increase in Bcl-2 and PPAR*γ* protein level of H9c2 cells were counteracted by the treatment with GW9962 (Figure 4(e)). In terms of ROS production and MMP, GW9962 disrupted the *β*-sitosterol-induced decrease in ROS production and increase in the MMP of H/R-stimulated H9c2 cells (Figures 4(f) and 4(g)).

### 3.5. *β*-Sitosterol Treatment Alleviated Myocardial I/R Injury in the Mice

**Figure 5:**
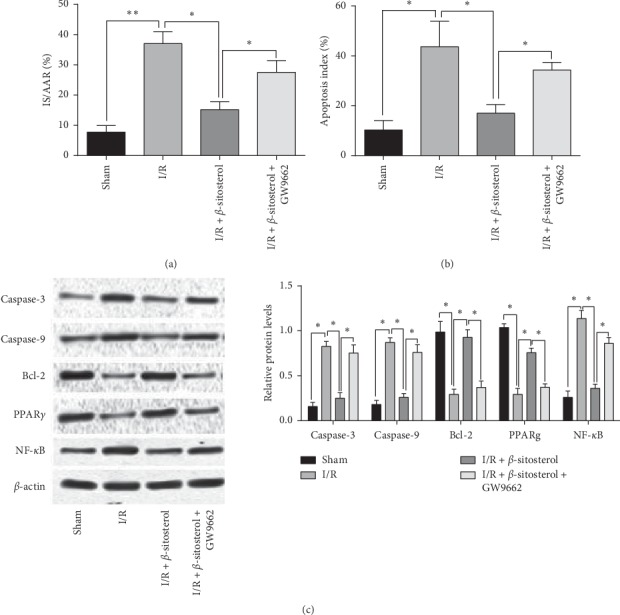
*β*-Sitosterol treatment alleviated I/R injury in the mice. (a) Infarcted size of the heart tissues in I/R mice subjected to *β*-sitosterol and/or GW9962 treatment. (b) Cell apoptotic rates of the heart tissues in I/R mice subjected to *β*-sitosterol and/or GW9962 treatment. (c) Protein levels of caspase-3, -9, Bcl-2, PPAR*γ*, and NF-*κ*B in the heart tissues of I/R mice subjected to *β*-sitosterol and/or GW9962 treatment. *N* = 6. ^*∗*^*P* < 0.05 and ^*∗∗*^*P* < 0.01.

The protective effects of *β*-sitosterol on the myocardial I/R injury were evaluated in the mouse. The I/R mouse showed the increased infarcted area and cell apoptosis in the heart tissues (Figures 5(a) and 5(b)), and *β*-sitosterol treatment reduced the infarcted area and cell apoptosis of the heart tissues from the I/R mouse (Figures 5(a) and 5(b)). GW9962 treatment impaired the protected effects of *β*-sitosterol against I/R injury in the mouse hearts (Figures 5(a) and 5(b)). Additionally, the protein levels of caspase-3, -9, and NF-*κ*B were increased, and protein levels of Bcl-2 and PPAR*γ* were decreased in the heart tissues from I/R mice, which was attenuated by the administration of *β*-sitosterol. Furthermore, the *β*-sitosterol-mediated decrease in the protein levels of caspase-3, -9, and NF-*κ*B and increase in the protein levels of Bcl-2 and PPARγ in I/R hearts were counteracted by the treatment with GW9962 (Figure 5(c)).

## 4. Discussion

Myocardial I/R injury is a complicated pathologic process, and the molecular mechanisms underlying this process remain unclear [11]. *β*-Sitosterol has been shown to be protective against I/R injury in different disease conditions [7, 9, 10, 12–16]. In the present study, we showed that OGD/R treatment suppressed cell viability and induced cell apoptosis and ROS production but reduced MMP, and *β*-sitosterol treatment attenuated H/R-induced cardiomyocyte injury, which was consistent with previous findings showing that *β*-sitosterol exerted protective actions against oxidant injury in H9c2 cells and rat hearts [7]. However, the signalling pathway that can be potentially affected by *β*-sitosterol was still elusive. Thus, in a further mechanistic study, our study for the first time showed that *β*-sitosterol treatment counteracted the inhibitory effects of H/R treatment on the PPAR*γ* protein expression and the enhanced effects of H/R treatment on the NF-*κ*B protein expression in H9c2 cells. Furthermore, inhibition of the PPAR*γ*/NF-*κ*B signalling impaired the protective actions of *β*-sitosterol against H/R-induced cardiomyocyte injury. In the I/R rats, *β*-sitosterol treatment reduced the myocardial infarcted size and apoptosis, which was attenuated by the inhibition of PPAR*γ*. Collectively, our results implied that *β*-sitosterol exerted the protective actions against myocardial I/R injury via modulating the PPARγ/NF-*κ*B signalling.

*β*-Sitosterol belongs to a class of phytosterols and exhibits multiple biological functions. *β*-Sitosterol was effective to block the hypoxia/reoxygenation-increased activation of protein tyrosine kinase in endothelial cells [17, 18]. *β*-Sitosterol is one of the components of the *N. deflersiana* ethanolic extract, which exhibited strong antioxidant, cardioprotective, anti-inflammatory, and antiapoptotic potential against isoproterenol-induced myocardial injury [8]. *β*-Sitosterol exerted protective actions against carbon tetrachloride hepatoxicity in the rats via improving the ability of mitochondrial function in the rat livers [9]. In addition, simvastatin, *β*-sitosterol, and punicalagin cotreatments could significantly reduce the ROS production by more than 70% in the macrophages [19]. In terms of MMP, *β*-sitosterol could increase the mitochondrial membrane potential via promoting the inner mitochondrial membrane fluidity [20]. Consistently, our results showed that *β*-sitosterol concentration dependently promoted cell viability, inhibited cell apoptosis and ROS production, and increased MMP in H/R-treated H9c2 cells, indicating that *β*-sitosterol may exert protective effects against H/R-induced cardiomyocyte injury via reducing oxidative stress and promoting mitochondrial function.

As *β*-sitosterol treatment could increase the PPAR*γ* expression in the irradiated rats [21], we further determined if *β*-sitosterol could affect the PPAR*γ* expression. We found that PPAR*γ* protein expression level was markedly decreased in H/R-treated H9c2 cells, while *β*-sitosterol treatment increased PPAR*γ* protein expression levels in the H/R-treated H9c2 cells. PPAR*γ* belongs to a class of nuclear receptors and plays a key role in the energy substrate metabolism and inflammatory responses [22]. Several studies have shown that activation of PPAR*γ* exhibited protective effects against myocardial I/R injury in the mice and rats. Khandooudi N et al. showed that the PPAR*γ* activator exerted the protective effects against myocardial I/R injury via inhibiting Jun NH2-terminal kinase/activating protein 1 [23]. Recent evidence showed that activation of PPAR*γ* could exert the antiapoptotic and anti-inflammatory function via repressing NF-*κ*B activity during myocardial I/R injury [24]. In a consistent manner, *β*-sitosterol was effective to increase the PPARγ protein expression but inhibited the NF-*κ*B expression in the H/R-treated cardiomyocytes. Furthermore, inhibition of PPAR*γ* impaired the protective actions of *β*-sitosterol against H/R-induced H9c2 cell injury. More importantly, the protective actions of *β*-sitosterol against myocardial I/R injury were verified in the rat model. Taken together, *β*-sitosterol-mediated myocardial protective effects may involve the modulation of PPAR*γ*/NF-*κ*B signalling.

In conclusion, our results demonstrate that *β*-sitosterol protected against *in vitro* H/R-induced cardiomyocyte injury and *in vivo* myocardial I/R injury. The *β*-sitosterol-mediated cardioprotective effects may involve the modulation of PPAR*γ*/NF-*κ*B signalling during myocardial I/R injury. *β*-Sitosterol is widely distrusted in the vegetable oil, nuts, and prepared foods, and a diet supplemented with natural *β*-sitosterol can be advised to potentially reduce the risk of myocardial injury. However, further studies are required to further explore the clinical application of *β*-sitosterol in the myocardial I/R injury.

## Data Availability

All data used to support the findings of this study are including within the article.
